# Effects of Designed Herbal Formula on Growth Performance, Blood Indices, Organ Traits, and Cecum Microbiology in Broilers

**DOI:** 10.3390/vetsci11030107

**Published:** 2024-02-29

**Authors:** Yuelong Sun, Mengjie Zhang, Dongdong Shi, Xiaofeng Dai, Xiumei Li

**Affiliations:** Key Laboratory of Feed Biotechnology, Ministry of Agriculture and Rural Affairs, Institute of Feed Research of CAAS, Beijing 100081, China

**Keywords:** designed herbal formula, growth performance, blood indices, organ traits, cecum microbiology, broilers

## Abstract

**Simple Summary:**

Antibiotics have long played a positive role in enhancing broiler growth performance and improving intestinal health. However, the long-term use of antibiotics has led to problems such as resistance, which has serious implications for global public health. Herbs are considered ideal as new antibiotic alternatives because they are natural, non-resistant, and non-toxic or have low toxicity. However, the application of herbal formulas in broiler farming is still relatively rare. Therefore, the aim of this study was to investigate the effects of the designed herbal formula (*Astragali radix*, *Atractylodes macrocephala* Koidz., *Isatis tinctoria* Linnaeus, and *Citri reticulatae pericarpium*) on growth performance, blood indices, organ traits, and cecum microbiology in broilers. The results demonstrated that the herbal formulas played a positive role in enhancing growth performance, immune performance, and cecum microbiological composition in broilers. Our study provides a theoretical basis and practical experience for the application of herbs in poultry farming and help to develop them as novel feed additives.

**Abstract:**

The objective of this study was to investigate the effect of the designed herbal formula (DHF) on growth performance, blood indices, organ traits, and cecum microbiology in broilers. A total of 96 male broilers of 1 d were selected and randomly assigned to two groups with six replicates of eight broilers each. The control (CON) and the basal diet containing 1.0% DHF (*Astragali radix*, *Atractylodes macrocephala* Koidz., *Isatis tinctoria* Linnaeus, and *Citri reticulatae pericarpium*, 2:1:1:2) were fed separately. The experiment was conducted for 35 days. The results showed that the DHF diet increased body weight and decreased the feed conversion ratio (FCR) (*p* < 0.05). At 21 days, the spleen, thymus, lymphocytes, and thrombocytes were increased (*p* < 0.05), and pancreas, duodenum, heterophils, and mean corpuscular hemoglobin (MCH) were decreased (*p* < 0.05). At 35 days, the heart, pancreas, white blood cell, heterophils, hemoglobin, MCH and mean corpuscular hemoglobin concentration (MCHC) were decreased, while lymphocytes and middle cells were increased (*p* < 0.05). The results of microbial diversity analysis showed that the DHF diet decreased the microbial diversity of the cecum. Firmicutes and Bacteroidetes were the dominant phyla, where the DHF diet increased the relative abundances of *Bacteroides uniformis*, *Bacteroides vulgatus*, and *Faecalibacterium prausnitzii*, and then decreased the relative abundance of *Shigella sonnei*. In conclusion, DHF played a positive role in improving the growth performance, immune performance, and relative abundance of *Bacteroides uniformis*, *Bacteroides vulgatus*, and *Faecalibacterium prausnitzii* in cecum microbiology in broilers, and has the potential to be used as a novel feed additive.

## 1. Introduction

In recent years, the global poultry industry has grown rapidly. Specifically, broilers account for a large proportion of global meat consumption, with advantages such as a lower cost of production and shorter growth cycles [1]. The intensive and large-scale development of broiler farming may bring problems such as decreased growth performance, frequent diseases, and intestinal dysfunction in broilers [2]. For a long time, antibiotics has played a positive role in improving the growth performance of broilers, reducing the incidence of diseases, and improving gut health [3]. However, the long-term use of antibiotics has led to problems such as drug resistance, which has a serious impact on global public health [4,5,6]. The ban on antibiotics in feed has forced people to look for new antibiotic alternatives to ensure the healthy growth of broilers.

Currently, herbal medicines, probiotics, prebiotics, peptides, and enzymes are considered good alternatives to antibiotics [7,8,9,10,11]. Among them, herbal medicine is considered ideal for new antibiotic substitutes because of its naturalness, non-resistance, non-toxicity, or low toxicity [12]. For example, *Astragali radix* contains a variety of compounds, such as polysaccharides, saponins, flavonoids, and amino acids, which have a positive regulatory effect on the development of immune organs and intestinal microorganisms in poultry [13]. *Atractylodes macrocephala* Koidz. is rich in a variety of biological components, such as terpenoids, coumarins, flavonoids, steroids, and polysaccharides, and has been shown to promote the proliferation of peripheral lymphocytes in broilers with strong immune-enhancing activity [14]. A mixture of *Isatis tinctoria* Linnaeus (main components include indoles, alkaloids, and glycosides) and other drugs play a positive role in the antipyretic effect on lipopolysaccharide-induced febrile broilers by inhibiting the TLR4/NF-κB signaling pathway and the activation of thermogenic factors [15]. *Citri reticulatae pericarpium* is rich in flavonoids, such as hesperidin, which has good antioxidant and anti-inflammatory activities, and can improve the growth performance of broilers and positively regulate the antioxidant defense performance of broilers [16]. There is enough experience in the use of herbal medicine as feed additives in the breeding of broilers and other poultry [17,18]. This has not been limited to a single herb as part of the dietary composition, but has also included herbal formulas consisting of more than one herb [19]. Often, herbal formulas are more therapeutic than single herbs. By combining different herbs, they can exert antioxidant, anti-inflammatory, growth-promoting, and many other activities. For example, in a liver-protection test in a broiler model of acute liver injury, the herbal formula (*Capillaries*, *Gentian*, *Gardenia*, *Bupleurum* root, and *Licorice*) had a better hepatoprotective effect on carbon tetrachloride-induced acute liver injury [20]. In a trial with coccidia-infected broilers, the herbal formula (*Cnidium monnieri*, *Taraxacum officinale*, and Sodium chloride) alleviated pathological changes in organs such as the cecum and spleen, increased the immune organ index, and modulated the gut microbiological composition of broilers by increasing the ratio of *Lactobacillus* and other bacteria [21]. Despite the results on the use of herbal formulas in broiler research, few studies have been conducted on herbal formulas consisting of *Astragali radix*, *Atractylodes macrocephala* Koidz., *Isatis tinctoria* Linnaeus, and *Citri reticulatae pericarpium* in broilers.

Therefore, the present study was proposed to investigate the effects of a designed herbal formula (DHF, *Astragali radix*, *Atractylodes macrocephala* Koidz., *Isatis tinctoria* Linnaeus, and *Citri reticulatae pericarpium*) obtained from the previous studies by our laboratory on growth performance, blood indices, organ traits, and cecum microbiology in broilers by adding them to broiler diets. Our study will provide a theoretical basis and practical experience for the application of herbal medicine in poultry farming and can help to develop it as a novel feed additive.

## 2. Materials and Methods

### 2.1. Herbal Formula Preparation

The formula consisted of *Astragali radix* (dried root), *Atractylodes macrocephala* Koidz. (dried root), *Isatis tinctoria* Linnaeus (dried root), and *Citri reticulatae pericarpium* (dried ripe pericarp of *Citrus reticulata* Blanco and its varieties) at a ratio of 2:1:1:2. This ratio was obtained by our laboratory in a previous study. The herbs were crushed into powders and mixed. All the herbs were purchased from Hebei Anguo Traditional Chinese Medicine Market (Anguo, China).

### 2.2. Animals, Diets, and Experimental Design

The experiment was carried out according to procedures approved by the Animal Ethics Committee of the Institute of Feed Research, Chinese Academy of Agricultural Sciences (IFR-CAAS-20210528). The broilers used in this study were white-feathered broilers purchased from a nearby farm.

The addition of the formula was determined before the start of the formal experiment. A total of 160 male healthy Arbor Acres (AA) broilers at 1 d with similar body weights (39.64 ± 0.27) were randomly assigned to 4 groups with 4 replicates of 10 broilers each. The control group (CON) was fed the basal diet and the experimental group was fed DHF added at 0.5%, 1.0%, and 1.5%. DHF was supplemented in the form of dry powder during the preparation of the diets, which were mixed thoroughly and stored. The experimental period was 21 days. At the end of the experiment, the optimum addition was selected according to the broiler body weight. The composition and nutrient content of the basal diet are shown in Table S1. All broilers were raised in cages (length × width × height, 195 × 68 × 66 cm) that could accommodate 10 broilers, all of which were allowed to eat and drink freely.

A total of 96 male healthy Arbor Acres (AA) broilers at 1 d with similar body weights (43.37 ± 0.16) were randomly assigned to 2 groups with 6 replicates of 8 broilers each. The control (CON) group was fed a basal diet, and the DHF group was fed a basal diet with 1% DHF. DHF was supplemented in the form of a dry powder during the preparation of diets, which was mixed thoroughly and stored. Diets were provided in two phases. The experimental diets were formulated to meet the minimum requirements of the National Research Council guidelines [22]. The composition and nutrient content of the basal diet are shown in Table 1. All broilers were raised in cages (length × width × height, 195 × 68 × 66 cm) that could accommodate 8 broilers, all of which were allowed to eat and drink freely. Regular cleaning and disinfection of chicken coops were performed.

### 2.3. Growth Performance

The broilers were weighed on days 21 and 35 of the experimental period and feed intake was recorded. The average daily weight gain (ADG), average daily feed intake (ADFI), and feed conversion ratio (FCR) were calculated according to the previously described method [23].

### 2.4. Organ Traits

On the 21st day of the test period, one broiler was randomly selected from each replicate of each group and slaughtered in accordance with the Operating Procedures for Livestock and Poultry Slaughtering (GB/T 194728-2018) [24]. The internal organs (heart, liver, stomach, pancreas, duodenum, and cecum) and immune organs (spleen, bursa of Fabricius, and thymus) were weighed. The internal organ and immune organ traits were the ratio of organ weight to the broiler body weight. Calculation of the internal organ and immune organ traits at 35 days followed the 21 days procedure.

### 2.5. Routine Blood Test

On the 21st day of the experimental period, one broiler was selected from each replicate in each group and 5 mL blood samples were obtained by subterminal venous blood collection. Broiler blood was analyzed using a Tek-ii automatic animal blood analyzer (Tecom Science Corporation, Nanchang, China). It mainly included white blood cells, lymphocytes, the percentage of lymphocytes, middle cells (monocytes, eosinophils, and basophils), and the percentage of middle cells associated with the immune response, and other blood indices. Blood indices at 35 days were determined by the same method.

### 2.6. Microbial Diversity of the Cecum

On the 35th day of the experimental period, one broiler with a similar body weight from each replicate in each group was selected and slaughtered, and the contents of the cecum were collected in sterile 5 mL freezing tubes and preserved on dry ice. The cecum microbial DNA was extracted using a TGuide S96 DNA Kit (Tiangen Biotechnology (Beijing) Co., Ltd., Beijing, China) and the procedure was carried out according to the kit’s instructions. The concentration of extracted DNA was quantified using the Qubit dsDNA HS Assay Kit and Qubit 4.0 Fluorometer (Thermo Fisher Scientific, Waltham, MA, USA). The full-length sequence of the 16S rRNA gene was amplified using 27F: AGRGTTTGATYNTGGCTCAG and 1492R: TASGGHTACCTTGTTASGACTT. To reduce the generation of chimeras in the amplification, specific PacBio sequences were added to the tail end of each primer. The PCR reaction was carried out on KOD One PCR Master Mix (Toyobo (Shanghai) Biotech Co., Ltd., Shanghai, China) with an initial denaturation temperature of 95 °C for 2 min, followed by denaturation at 98 °C for 10 s, annealing at 55 °C for 30 s, and extension at 72 °C for 1 min and 30 s for a total of 25 cycles. The final step was extension at 72 °C for 2 min. The amplification products were purified by Agencourt AMPure XP Beads (Beckman Coulter, Indianapolis, IN, USA) and quantified using a Qubit dsDNA HS Assay Kit and Qubit 4.0 Fluorometer (Thermo Fisher Scientific, Waltham, MA, USA). After the individual quantification step, amplicons were pooled in equal amounts. SMRTbell libraries were prepared with the SMRTbell Express Template Prep Kit 2.0 (Pacific Biosciences, Menlo Park, CA, USA). Purified SMRTbell libraries from the pooled and barcoded samples were sequenced on a single PacBio Sequel II 8M cell using the Sequel II Sequencing kit 2.0 (Pacific Biosciences, Menlo Park, CA, USA).

Bioinformatics analyses for this study were performed with the help of BMK Cloud (Biomarker Technology Co., Ltd., Beijing, China). Raw reads generated by sequencing were filtered and demultiplexed with a minimum pass rate of ≥5 and a minimum prediction accuracy of ≥0.9 using SMRT Link software (version 8.0, https://www.pacb.com/smrt-link/, accessed on 19 June 2022) to obtain circular consensus sequencing (CCS) reads. Subsequently, CCS sequences were assigned to the appropriate samples based on barcodes using lima (version 1.7.0, https://lima.how/, accessed on 19 June 2022). CCS reads without primers and CCS reads with lengths out of range (1, 200-1, 650 bp) were rejected by identifying forward and reverse primers and quality filtering using the Cutadapt [25] quality control process (version 2.7, http://cutadapt.readthedocs.org/, accessed on 19 June 2022). The UCHIME [26] algorithm (version 8.1, http://drive5.com/uchime/uchime_download.html, accessed on 19 June 2022) was used to detect and remove chimeric sequences to obtain clean reads. Sequences with ≥97% similarity were clustered into identical operational taxonomic units (OTUs) by USEARCH (version 10.0, http://drive5.com/usearch, accessed on 19 June 2022) and OTUs with <0.005% heavy abundance were filtered [27]. OTUs were classified and annotated using the SILVA [28] database (version 132, https://www.arb-silva.de/, accessed on 19 June 2022) with 70% confidence based on the Naive Bayes classifier in QIIME2 (version 2020.6.0, https://qiime2.org, accessed on 19 June 2022) [29]. The alpha diversity was calculated and displayed using QIIME2 and R software, respectively. Beta diversity was determined using QIIME to assess the degree of similarity of microbial communities across samples. Partial least squares discrimination analysis (PLS-DA) was performed using the R software and mixOmics (v6.3.2, https://CRAN.R-project.org/package=mixOmics, accessed on 19 June 2022) [30]. Non-metric multidimensional scaling (NMDS) was determined using QIIME. In addition, linear discriminant analysis (LDA) for effect size (LEfSe, version 1.1.1, https://github.com/SegataLab/lefse/tree/master/lefse, accessed on 19 June 2022) was used to test for significant categorical differences between groups [31]. A log LDA score of 3.0 was set as the threshold for discriminant features.

### 2.7. Statistical Analysis

Data from the pre-experiment were analyzed using one-way analysis of variance (ANOVA) using SPSS (version 26.0, https://www.ibm.com/cn-zh/spss, accessed on 24 January 2024). Post hoc tests were performed using the LSD method. The data from the formal experiment were analyzed using the general linear model of two-way analysis of variance (ANOVA), with day and diet as the dominant effects. When the chi-square test presented significance (*p* < 0.05), the analysis was performed using the Welch test. The results were presented as the “mean ± SD” and differences between groups were considered significant when *p* < 0.05. Microbial diversity analysis was performed using BMKCloud (www.biocloud.net, accessed on 19 June 2022). The Shannon and Chao 1 indices were analyzed using Student’s *t* test. The sample distance algorithm in NMDS analysis was binary-Jaccard. The species composition at different taxonomic levels was reported as the relative abundance. Different species with LDA scores greater than 3.5 in LEfSe analysis were considered statistically distinct biomarkers.

## 3. Results

### 3.1. Pre-Experimental Growth Performance

As shown in Table 2, the 1.0% DHF significantly increased body weight and ADG and significantly decreased feed conversion ratio at the 21st day (*p* < 0.05). The 0.5 and 1.5% DHF treatments did not affect broiler growth performance (*p* > 0.05). Meanwhile, all the added ratios of DHF did not significantly affect the feed intake of broilers (*p* > 0.05). Therefore, 1.0% was optimal.

### 3.2. Growth Performance

As shown in Table 3, time and diet significantly affected the changes in body weight, ADG, and FCR (*p* < 0.05). Within 1–21 days, the DHF diet significantly increased ADG and body weight (*p* < 0.01) and significantly decreased FCR (*p* < 0.01). Within 1–35 days, the DHF diet significantly increased ADG and body weight, and decreased FCR (*p* < 0.01). In addition, time significantly affected ADFI (*p* < 0.05), while the effect of diet on ADFI was not significant (*p* > 0.05).

### 3.3. Organ Traits

As shown in Table 4, differences in the heart, bursa of fabricius, pancreas, thymus, and stomach were caused by diet and time (*p* < 0.05). The difference for the duodenum was only related to diet (*p* < 0.05). The differences for the liver and spleen were only related to time (*p* < 0.05). The cecum was not affected by time and diet (*p* > 0.05). On day 21 of the experimental period, the DHF diet significantly increased the thymus index (*p* < 0.05) and decreased the pancreas index and duodenum index (*p* < 0.05). The DHF diet had no significant effect on other visceral and immune organ indices (*p* > 0.05). On day 35 of the experimental period, the DHF diet significantly decreased the heart index, pancreas index, and stomach index (*p* < 0.05), with no significant effect on the other visceral and immune organ indices (*p* > 0.05).

### 3.4. Routine Blood Test

As shown in Table 5, changes in lymphocytes, middle cells (%), and MCHC were only related to diet (*p* < 0.05). The changes in white blood cells, heterophils, lymphocytes (%), and MCH were correlated with diet and time (*p* < 0.05). On day 21 of the experimental period, the DHF diet significantly increased the content of lymphocytes (including the percentage content) and significantly decreased the contents of heterophils and MCH (*p* < 0.05). On day 35 of the experimental period, the DHF diet significantly increased the contents of lymphocytes (including the percentage content) and middle cells in plasma, and significantly decreased the contents of white blood cells, heterophils, MCH, and MCHC (*p* < 0.05), and had no significant effect on the other indices in plasma (*p* > 0.05).

### 3.5. Microbial Diversity of the Cecum

To further investigate the effect of the DHF on the gut microorganisms, the cecum contents of broilers were analyzed for 16S rRNA. A total of 163,895 CCS (circular consensus sequencing) and 161,009 CCS sequences were obtained after quality control, with a sequence validity of 98.25% and an average sequence length of 1458 bp. These sequences were clustered into 844 OTUs based on 97% similarity. There were 667 OTUs in the two groups, with 91 feature OTUs in the CON group and 91 feature OTUs in the DHF group. The DHF decreased the feature OTUs in broilers compared with the CON group.

#### 3.5.1. Alpha and Beta Diversity Analysis

**Figure 1:**
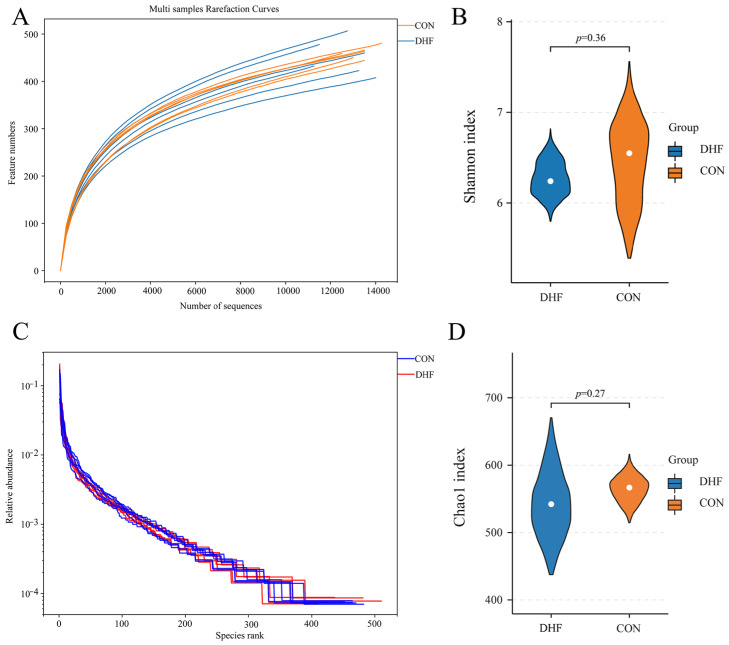
Analysis of alpha diversity among different groups. (**A**): Sample dilution curve. (**B**): Shannon index of cecum microorganisms. (**C**): Species accumulation curve. (**D**): Chao 1 index of cecum microorganisms. CON: control. DHF: designed herbal formula.

**Figure 2:**
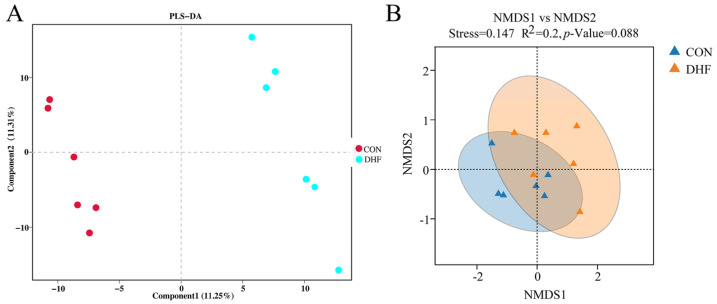
Analysis of beta diversity among different groups. (**A**): PLS-DA analysis of cecum microorganisms. (**B**): NMDS analysis of cecum microorganisms. CON: control. DHF: designed herbal formula.

As shown in Figure 1A, the rarefaction curve gradually flattened as the sample size increased, and no large number of species was found, indicating that the sample sequence of this experiment was sufficient to meet the requirements of data analysis. Meanwhile, the rank abundance curve indicated that the current sample had good richness and evenness (Figure 1C). Alpha diversity analysis showed that the DHF decreased the Shannon and Chao 1 indices (Figure 1B,D). The DHF decreased the number of microbial species in the cecum of broilers. The results of beta diversity analysis showed a greater degree of dispersion between the two groups (Figure 2A,B). The DHF was the main factor affecting the microbial species diversity of the broiler cecum.

#### 3.5.2. Analysis of Community Composition

**Figure 3:**
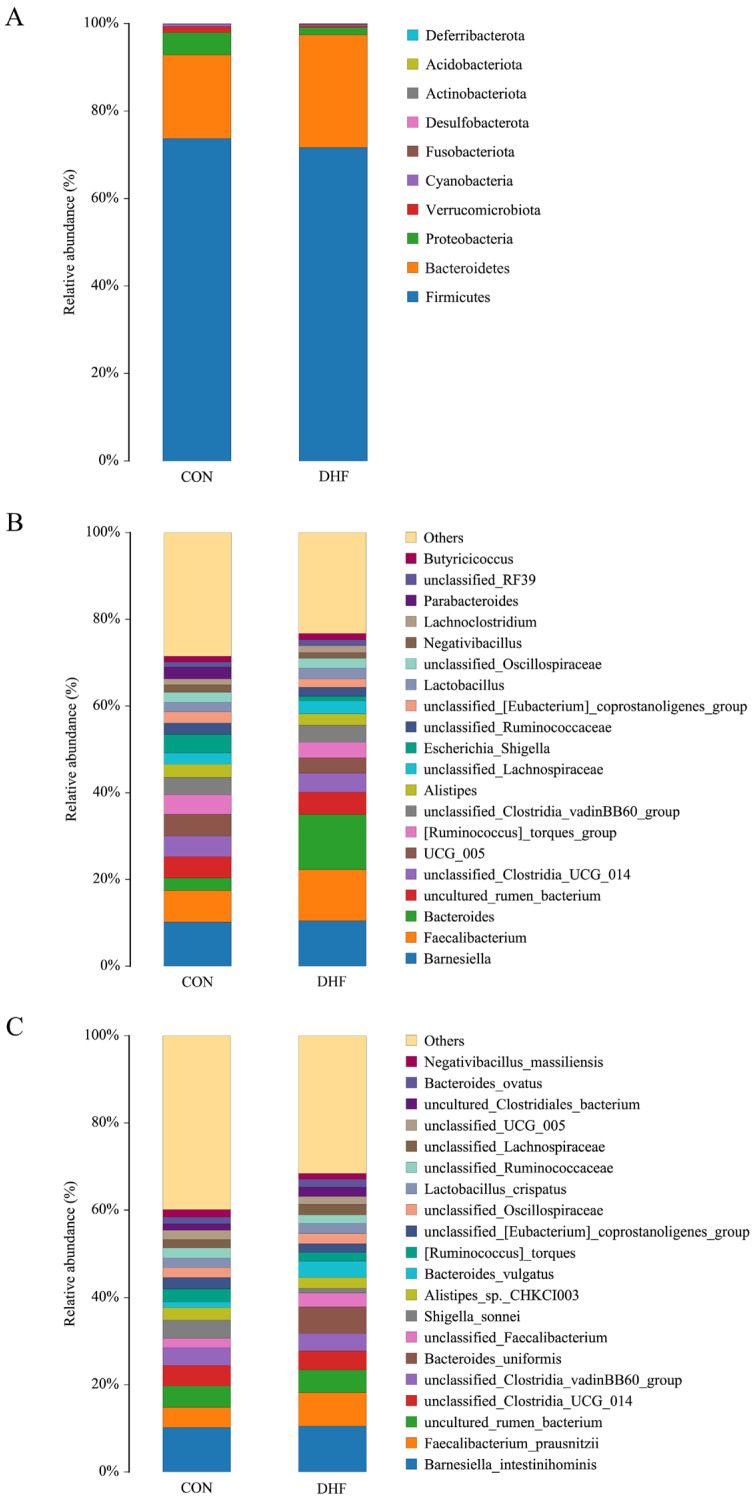
Taxonomic composition of cecum microorganisms among different groups. (**A**) Species composition at the phylum level. (**B**) Species composition at the genus level. (**C**) Species composition at the species level. CON: control. DHF: designed herbal formula.

In order to clarify the effect of the DHF on cecum microbial species diversity in broilers, the species composition of cecum microorganisms was further analyzed. At the phylum level, the species composition was as follows: Firmicutes, Bacteroidetes, Proteobacteria, Verrucomicrobiota, Cyanobacteria, Acidobacteriota, Desulfobacterota, Actinobacteriota, Fusobacteriota, Gemmatimonadota, and others (Figure 3A). Among them, the dominant phyla in both groups were Firmicutes and Bacteroidetes. The DHF decreased the abundance of Proteobacteria and Firmicutes, and increased the abundance of Bacteroidetes.

At the genus level, the top 20 cecum microorganisms by relative abundance are shown in Figure 3B to be *Barnesiella*, *Faecalibacterium*, *Bacteroides*, uncultured rumen bacterium, unclassified *Clostridia* UCG 014, UCG 005, *[Ruminococcus] torques* group, unclassified *Clostridia vadin* BB60 group, *Alistipes*, unclassified *Lachnospiraceae*, *Escherichia-Shigella*, unclassified *Ruminococcaceae*, unclassified *[Eubacterium] coprostanoligenes* group, *Lactobacillus*, unclassified *Oscillospiraceae*, *Negativibacillus*, *Lachnoclostridium*, *Parabacteroides*, unclassified RF39, *Butyricicoccus*, and Others. The DHF increased the relative abundances of *Bacteroides*, *Lactobacillus,* and *Faecalibacterium* and decreased the relative abundance of *Escherichia-Shigella*. Notably, *Parabacteroides* was not detected in the DHF group.

At the species level, the top 20 cecum microorganisms in terms of relative abundance are shown in Figure 3C to be *Barnesiella intestinihominis*, *Faecalibacterium prausnitzii*, uncultured rumen bacterium, unclassified *Clostridia* UCG 014, unclassified *Clostridia vadin* BB60 group, *Bacteroides uniformis*, unclassified *Faecalibacterium*, *Shigella sonnei*, *Alistipes* sp. CHKCI003, *Bacteroides vulgatus*, *[Ruminococcus] torques*, unclassified *[Eubacterium] coprostanoligenes* group, unclassified *Oscillospiraceae*, *Lactobacillus crispatus*, unclassified *Ruminococcaceae,* unclassified *Lachnospiraceae*, unclassified UCG 005, uncultured *Clostridiales bacterium*, *Bacteroides ovatus*, *Negativibacillus massiliensis*, and others. The DHF increased the relative abundances of *Bacteroides vulgatus*, *Bacteroides uniformis*, and *Faecalibacterium prausnitzii* and decreased the relative abundance of *Shigella sonnei*.

#### 3.5.3. Analysis of Significance of Differences between Groups

**Figure 4:**
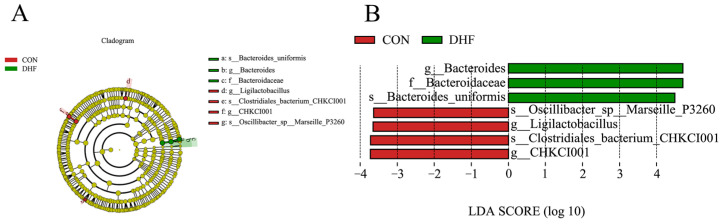
LEfSe analysis between groups. (**A**) LEfSe analysis of evolutionary branching diagrams. (**B**) Histogram of LDA value distribution. CON: control. DHF: designed herbal formula.

LEfSe analysis showed significant differences between the two groups (Figure 4). As shown in Figure 4A, compared with the CON group, Bacteroidaceae, *Bacteroides*, and *Bacteroides uniformis* were enriched in the DHF group, while *Oscillibacter* sp. *Marseille* P3260, *Ligilactobacillus*, CHKCI001, and *Clostridiales bacterium* CHKCI001 were enriched in the CON group. In addition, the relative abundance of *Bacteroides uniformis* was higher than that of the other species and could be recognized as a biomarker that contributed significantly to the difference between the two groups (Figure 4B). In conclusion, the DHF improved the microbial structural composition of the broiler cecum, reduced the microbial species diversity, promoted the growth of beneficial bacteria, and inhibited the multiplication of harmful bacteria.

## 4. Discussion

It is well known that, with the extensive use of antibiotics (growth promoters, etc.) in the poultry industry, problems such as resistance have arisen [32]. This has forced many countries around the world to work on developing new antibiotic alternatives. Herbal medicine, as a natural product, is safe, effective, has low toxicity, is non-residual, and does not easily produce drug resistance, making it one of the best choices for antibiotic substitutes [33,34]. In the present study, the DHF had a significant positive effect on broiler growth performance. At 21 days, the ADG increased by 11.12%, body weight increased by 9.02%, and FCR decreased by 7.14%. In addition, at 35 days, the ADG increased by 7.17%, body weight increased by 7%, and FCR decreased by 4.49%. Such results were in line with the findings of several previous studies that herbs could improve the growth performance of broilers [35,36,37,38]. The application of herbal medicines as feed additives in broiler farming has more experience to be found and has shown better application prospects. For example, adding 0.05% chicory (*Cichorium intybus* L.) to the diet increased the body weight and feed intake in broilers at 21 and 35 days [39]. *Ocimum gratissimum* dietary additives (5 g/kg) increased broiler growth performance and improved the immune response of broilers under high-temperature conditions [40]. The addition of licorice could promote the development of poultry organs, improve growth performance, and maintain body health [41]. Therefore, the use of these natural plants, which are rich in bioactive substances, as feed additives may be one of the best choices. It is worth noting that the current application of herbs as feed additives is mainly based on single herbs, and the application of mixed herbs as feed additives is relatively small. From a medical point of view, the use of herbal medicine is mainly based on a variety of herbs, and the efficacy of different herbs varies. Targeting the diseased area, multiple herbs can work simultaneously to achieve the purpose of treatment or improvement [42]. Although single herbs have made good progress in broiler farming, the advantages of mixed herbs are still noteworthy. As far as we can see, mixed herbs have the potential to modulate broiler growth performance and improve the skeletal characteristics of broilers [43]. Lipiński et al. [35] reported that the addition of mixed herbs to broiler diets improved the growth performance, carcass traits, and meat quality. Ghafouri et al. [44] provided strong evidence that mixed herbs could play a positive role in broiler farming by feeding diets containing herbal mixtures.

As a novel alternative to antibiotics, herbs also play an active role in the immune performance of broilers [45]. In the present study, the DHF increased thymus indices at 21 days and remained essentially the same as CON at 35 days. The thymus is an important immune organ in poultry and plays an important role in maintaining the health of the body [46]. It has been shown that the weight of immune organs correlates with the strength of the body’s immune function [47]. It is worth noting that broilers are at a low level of immunocompetence at around 20 days and are susceptible to pathogenic bacteria. The DHF in this study was effective in increasing the immune organ index at 21 days, enhancing the immune ability of the organism and contributing to the protection of the organism’s health, which was similar to the results of previous studies [48]. In addition, the thymus, as an important immune organ, is a site of lymphocyte differentiation and maturation [49]. Further analysis of the blood of the test broilers showed that the DHF increased the number of lymphocytes, which was observed at both 21 and 35 days. This suggested that the herbal medicine promoted the development of immune organs and the differentiation of lymphocytes, which had a positive effect on the improvement of broiler immunity [50,51,52]. Meanwhile, the present study observed that, at 35 days, lymphocytes were still higher than those in the CON group, although the immune organ traits were the same as those of the CON group. This may be due to changes in gut microorganisms.

In recent years, gut microorganisms have often been used to explain the effects of external factors, such as environmental changes, on the host [53]. As important immune and neuroendocrine organs of the body, the role played by gut microorganisms cannot be ignored [54]. For this reason, we further analyzed the effects of the DHF on gut microorganisms to attempt to explain the effects of the DHF on broilers. In the present study, the DHF decreased the cecum microbial species diversity of broilers compared with the CON group, which was observed based on the decreases in the Shannon and Chao 1 indices. Additionally, the separation between groups, as demonstrated by the beta diversity analysis, suggested that the DHF affected the microbial composition of the broiler [55]. Further analysis of the microbial composition revealed that Firmicutes and Bacteroidetes were the dominant groups of cecum microorganisms [56], and the DHF decreased the relative abundances of Firmicutes and Proteobacteria and increased the relative abundance of Bacteroidetes.

At the genus level, DHF increased the relative abundances of *Bacteroides*, *Faecalibacterium*, and *Lactobacillus* and decreased the relative abundance of *Escherichia-Shigella*. As an important intestinal resident bacterium, *Bacteroides* can secrete a variety of metabolic enzymes, has metabolic capacity for polysaccharides, etc., and can deliver nutrients and beneficial metabolites to the host or other intestinal commensal bacteria, which can play an active role in maintaining intestinal homeostasis and preserving the stability of the immune system of the body [57]. *Faecalibacterium* is also an important intestinal commensal and is considered a biomarker of the health of the organism. Studies have shown that *Faecalibacterium* might be involved in the treatment or amelioration of a wide range of diseases and might maintain host health through the production of energy and anti-inflammatory metabolites [58]. Luo et al. [59] added dietary vitamins to broiler diets and showed that they could increase the relative abundance of *Faecalibacterium* and have a positive effect on broiler gut health. It is well known that *Escherichia-Shigella* is associated with intestinal inflammation and ecological disorders, and has been listed as a key genus to be observed by the poultry industry because it can produce a variety of enterotoxins, undermine the integrity of the intestinal barrier, and is prone to cause intestinal flora dysbiosis [60,61,62,63]. Herbs have long been prominent in the suppression of *Escherichia-Shigella*. Wang et al. [64] demonstrated that the addition of amaranth to the diet increased the relative abundances of *Bacteroides* and *Lactobacillus* and decreased the relative abundance of *Escherichia-Shigella* as a means of regulating intestinal microbial homeostasis in broilers. Similar results were obtained in this study.

At the species level, the DHF increased the relative abundances of *Bacteroides vulgatus*, *Bacteroides uniformis*, and *Faecalibacterium prausnitzii* and decreased the relative abundance of *Shigella sonnei*. This is in line with previous studies [65,66,67]. It is worth noting that the single row of *Bacteroides uniformis* was significantly enriched in this study and could be considered as a biomarker strain produced by the addition of the DHF. A recent study revealed a significant enrichment of carbohydrate-active enzymes in the genome of *Bacteroides uniformis*, which encodes a specific protein conferring the ability to degrade a wide range of glycans and possessing multiple glycosidase activities [68]. Surprisingly, this provides strong theoretical support for the utilization of herbal medicines by *Bacteroides uniformis*. In addition, relevant studies have demonstrated that *Bacteroides uniformis* plays an important role in enhancing immunity levels [69]. Notably, there is also a correlation between *Bacteroides uniformis* and host body weight [70]. At the same time, we observed an increase in the relative abundance of *Faecalibacterium prausnitzii* with the DHF. *Faecalibacterium prausnitzii* is an important butyrate-producing bacterium with anti-inflammatory properties, capable of maintaining enzyme activity and protecting the digestive system from pathogenic bacteria [71]. Studies have shown that *Faecalibacterium prausnitzii* could promote prebiotic fermentation, which could ferment simple sugars to produce butyrate, and it is important to note that butyrate promotes immune response modulation [72]. In addition, *Faecalibacterium prausnitzii* could produce salicylic acid, an important modulator in the inflammatory process [73]. Cesare et al. [74] found that *Faecalibacterium prausnitzii* and other probiotic abundances were increased after probiotics were added to broilers’ drinking water, suggesting a relationship with the health of the broiler. *Shigella sonnei* is an emerging global pathogen that is highly susceptible to bacterial diarrhea [75]. In recent years, *Shigella* has been found to be present in the intestines of broilers, as well by 16S rDNA sequencing [67]. Studies have shown that herbs exhibit better antibacterial activity against it. The study by Anokwuru et al. [76] provides strong evidence that herbs inhibit *Shigella sonnei*.

As mentioned above, the DHF showed a positive effect in this study. The underlying mechanism might be that DHF promoted the abundance of *Bacteroides uniformis* and *Faecalibacterium prausnitzii*, which are able to secrete a variety of digestive enzymes that contribute to the body’s ability to utilize nutrients, thereby improving growth performance. At the same time, they are able to participate in important immune responses, which might have been a potential factor in the enhancement of immune performance obtained in this study. In addition, the reduction in pathogenic bacteria might have provided assistance in the improvement of growth performance and immune performance. It was reasonable to assume that the changes in the relevant bacteria were brought about by the herbal compound. In conclusion, herbal medicine plays a very important role in regulating gut microbial homeostasis, which includes the promotion of probiotics and inhibition of pathogenic bacteria. Meanwhile, herbal medicine, as an alternative to antibiotics, as a feed additive has great prospects for improving growth performance, immunity level, and gut microbiology, but it is still important to note that the composition of herbal medicine is very complex, and their mechanism of action needs to be investigated in detail in order to ensure their smooth application in feed additives.

## 5. Conclusions

The results of the current study suggest that the addition of the designed herbal formula to the diet improved growth performance, immune performance, and improved gut microbial homeostasis in broilers. The designed herbal formula significantly increased body weight and average daily weight gain, and decreased the feed conversion ratio in broilers at 21 and 35 days compared with the control group; it also increased immune organ traits, such as the thymus as well as lymphocyte counts. In addition, the designed herbal formula increased the relative abundances of *Bacteroides uniformis* and *Faecalibacterium prausnitzii* and decreased the relative abundance of *Shigella sonnei*. In conclusion, the present study concludes that the designed herbal formula provides options for novel feed additives, but in-depth studies on the positive effects of the designed herbal formula on growth performance, immune performance, and cecum microbiology in broilers are needed to facilitate their development.

## Figures and Tables

**Table 1:** Ingredients and nutrient composition of the basal diet.

Ingredient	1~21 d		22~35 d	
	CON	DHF	CON	DHF
Corn (%)	54.75	53.75	57.68	56.68
Soybean meal (%)	37.00	37.00	33.30	33.30
Soya bean oil (%)	4.10	4.10	5.20	5.20
Calcium hydrogen phosphate (%)	1.35	1.35	1.40	1.40
Stone powder (%)	1.15	1.15	0.90	0.90
DHF (%)	-	1.00	-	1.00
Salt (%)	0.30	0.30	0.30	0.30
Methionine (%)	0.20	0.20	0.12	0.12
Lysine (%)	0.15	0.15	0.10	0.10
Premix ^1^ (%)	1.00	1.00	1.00	1.00
Total (%)	100.00	100	100	100
Metabolizable energy (kcal/kg)	3002.00	2971.36	3100.00	3069.36
Crude protein (%)	21.50	21.49	20.00	19.99
Lysine (%)	1.27	1.27	1.13	1.13
Methionine (%)	0.51	0.51	0.42	0.42
Tryptophan (%)	0.25	0.25	0.23	0.23
Arginine (%)	1.46	1.46	1.35	1.35
Threonine (%)	0.80	0.80	0.74	0.74
Valine (%)	0.98	0.98	0.92	0.92
Isoleucine (%)	0.87	0.87	0.81	0.81
Leucine (%)	1.75	1.74	1.65	1.64
Calcium (%)	1.00	1.00	0.90	0.90
Total phosphorus (%)	0.68	0.68	0.66	0.66
Non-phytate phosphorus (%)	0.45	0.45	0.40	0.40

^1^ Premix provided the following per kilogram of diet: VA 10,000.00 IU, VB_1_ 2.00 mg, VB_2_ 8.00 mg, VB_6_ 4.00 mg, VB_12_ 0.06 mg, VD_3_ 3,000.00 IU, VE 20.00 IU, VK_3_ 2.00 mg, biotin 0.20 mg, folic acid 1.00 mg, *D*-pantothenic acid 20.00 mg, nicotinic acid 50.0 mg, Cu (as copper sulfate) 10.00 mg, Fe (as ferrous sulfate) 60.00 mg, Mn (as manganese sulfate) 80.00 mg, Zn (as zinc sulfate) 60.00 mg, I (as potassium iodide) 0.20 mg, Se (as sodium selenite) 0.30 mg, and choline 1000 mg/kg.

**Table 2:** Effects of different additive ratios of DHF on growth performance of broilers.

Items	CON	0.5%DHF	1.0%DHF	1.5%DHF	SEM	*p*-Value
Initial weight (g)	39.58 ± 0.35	39.60 ± 0.18	39.75 ± 0.21	39.65 ± 0.39	0.07	0.843
21 day weight (g)	617.5 ± 47.05 ^b^	605 ± 15.56 ^b^	725.3 ± 50.61 ^a^	618 ± 76.17 ^b^	17.13	0.022
ADG (g)	27.51 ± 2.23 ^b^	26.92 ± 0.75 ^b^	32.98 ± 2.49 ^a^	27.54 ± 3.63 ^b^	0.85	0.016
ADFI (g)	43.93 ± 2.99	45.02 ± 1.88	47.09 ± 1.51	44.90 ± 2.93	0.62	0.349
FCR	1.60 ± 0.10 ^b^	1.62 ± 0.13 ^b^	1.43 ± 0.07 ^a^	1.64 ± 0.01 ^b^	0.05	0.049

Different superscript letters represent significant differences (*p* < 0.05), and the same letters or no letters represent no significant differences (*p* > 0.05). CON: control. DHF: designed herbal formula.

**Table 3:** Effects of DHF on growth performance of broilers.

Items	Groups		*p*-Value		
	CON	DHF	Time	Diet	Time × Diet
Body weight (g)			<0.001	<0.001	0.029
21 day	617.60 ± 16.40	673.28 ± 13.67 **		0.002	
35 day	1864.90 ± 61.80	1995.60 ± 19.04 **		0.004	
ADG (g)			<0.001	<0.001	0.541
21 day	27.35 ± 0.77	30.39 ± 1.10 **		0.006	
35 day	52.05 ± 1.76	55.78 ± 0.54 **		0.004	
ADFI (g)			<0.001	0.118	0.892
21 day	42.23 ± 1.82	43.33 ± 0.92		0.288	
35 day	95.18 ± 0.47	96.11 ± 1.21		0.263	
FCR			<0.001	<0.001	0.092
21 day	1.54 ± 0.03	1.43 ± 0.02 **		0.001	
35 day	1.78 ± 0.03	1.70 ± 0.02 **		0.004	

** Represents a significant difference from CON (*p* < 0.01). CON: control. DHF: designed herbal formula.

**Table 4:** Effects of DHF on organ traits (g/kg BW) of broilers.

Items	Groups		SEM	*p*-Value		
	CON	DHF		Time	Diet	Time × Diet
Heart				<0.001	0.040	0.253
21 day	5.64 ± 0.67	5.38 ± 0.76	0.20		0.543	
35 day	4.26 ± 0.72	3.40 ± 0.18 *	0.19		0.017	
Liver				<0.001	0.783	0.721
21 day	23.59 ± 2.34	23.16 ± 1.81	0.58		0.730	
35 day	18.69 ± 1.21	18.74 ± 0.71	0.27		0.924	
Spleen				<0.001	0.210	0.129
21 day	0.70 ± 0.13	1.00 ± 0.28 *	0.08		0.041	
35 day	1.48 ± 0.27	1.45 ± 0.30	0.08		0.858	
Bursa of fabricius				<0.001	0.037	0.055
21 day	4.07 ± 1.36	2.78 ± 0.52	0.34		0.056	
35 day	0.73 ± 0.12	0.67 ± 0.19	0.04		0.511	
Pancreas				<0.001	0.001	0.033
21 day	4.56 ± 0.45	3.67 ± 0.37 **	0.18		0.004	
35 day	2.58 ± 0.24	2.29 ± 0.13 *	0.07		0.027	
Thymus				<0.001	0.041	0.006
21 day	2.71 ± 0.44	4.08 ± 0.93 **	0.38		0.009	
35 day	2.03 ± 0.58	1.80 ± 0.47	0.15		0.753	
Stomach				<0.001	0.044	0.533
21 day	26.82 ± 3.79	23.01 ± 4.58	1.29		0.147	
35 day	15.90 ± 1.25	13.83 ± 2.87	0.69		0.151	
Duodenum				0.518	0.004	0.074
21 day	11.20 ± 1.88	8.46 ± 1.26 *	0.60		0.014	
35 day	9.85 ± 1.04	9.11 ± 0.74	0.27		0.188	
Cecum				0.365	0.067	0.964
21 day	6.96 ± 1.96	5.92 ± 0.51	0.42		0.234	
35 day	7.50 ± 1.56	6.41 ± 0.92	0.39		0.168	

* Represents a significant difference from CON (*p* < 0.05). ** Represents a significant difference from CON (*p* < 0.01). CON: control. DHF: designed herbal formula.

**Table 5:** Effects of DHF on plasma biological indices of broilers.

Items	Groups		SEM	*p*-Value		
	CON	DHF		Time	Diet	Time × Diet
White blood cells (10^9^/L)				<0.001	0.015	0.020
21 day	123.57 ± 2.22	123.32 ± 2.06	0.59		0.847	
35 day	140.61 ± 6.59	132.12 ± 3.41 *	1.93		0.019	
Lymphocytes (10^9^/L)				0.152	<0.001	0.056
21 day	60.20 ± 0.86	62.75 ± 1.13 **	0.47		<0.001	
35 day	59.66 ± 4.30	66.30 ± 1.98 **	1.36		0.006	
Middle cells (10^9^/L)				<0.001	0.353	0.724
21 day	17.79 ± 0.53	17.65 ± 0.70	0.17		0.713	
35 day	19.67 ± 0.39	19.37 ± 0.57	0.14		0.317	
Heterophils (10^9^/L)				<0.001	0.002	0.020
21 day	45.58 ± 2.16	42.92 ± 1.58 *	0.66		0.036	
35 d	61.28 ± 10.60	46.45 ± 4.38 *	3.16		0.010	
Lymphocytes (%)				0.009	0.001	0.112
21 day	48.37 ± 1.77	51.87 ± 1.60 **	0.70		0.005	
35 d	42.73 ± 4.99	50.33 ± 2.46 **	1.58		0.007	
Middle cells (%)				0.701	0.039	0.052
21 day	14.22 ± 0.26	14.23 ± 0.39	0.09		0.932	
35 day	13.95 ± 0.58	14.62 ± 0.19 *	0.16		0.023	
Heterophils (%)				0.015	<0.001	0.089
21 day	37.41 ± 1.90	33.90 ± 1.32 **	0.70		0.004	
35 day	43.32 ± 5.54	35.05 ± 2.55	1.72		0.013	
Red blood cells (10^12^/L)				0.397	0.633	0.139
21 day	2.46 ± 0.22	2.54 ± 0.10	0.05		0.421	
35 day	2.65 ± 0.25	2.49 ± 0.16	0.06		0.222	
Hemoglobin (g/L)				0.914	0.052	0.107
21 day	95.17 ± 8.84	94.00 ± 5.02	1.99		0.784	
35 day	100.00 ± 8.72	88.50 ± 6.72 *	2.76		0.028	
Hematokrit (L/L)				0.541	0.136	0.188
21 day	0.24 ± 0.02	0.24 ± 0.01	0.01		0.879	
35 day	0.26 ± 0.02	0.24 ± 0.02	0.01		0.095	
MCV (fL)				0.167	0.127	0.984
21 day	98.97 ± 1.63	97.00 ± 3.18	0.76		0.207	
35 day	97.20 ± 4.00	95.18 ± 2.96	1.01		0.344	
MCH (pg)				0.003	0.001	0.302
21 day	38.53 ± 0.43	37.15 ± 0.97 *	0.29		0.010	
35 day	37.62 ± 1.04	35.42 ± 1.16 **	0.45		0.006	
MCHC (g/L)				0.098	0.011	0.231
21 day	369.84 ± 7.20	364.17 ± 8.04	2.27		0.227	
35 day	368.00 ± 12.74	353.33 ± 3.31 *	3.54		0.030	
RDW-SD (fL)				0.154	0.067	0.233
21 day	12.76 ± 3.16	10.92 ± 0.13	0.62		0.183	
35 day	19.88 ± 12.31	11.58 ± 2.39	2.74		0.136	
RDW-CV (%)				0.860	0.756	0.953
21 day	30.68 ± 2.95	30.32 ± 0.79	0.60		0.774	
35 day	30.45 ± 3.35	30.20 ± 1.59	0.72		0.872	
Thrombocytes (10^9^/L)				<0.001	0.894	0.131
21 day	7.00 ± 2.10	10.17 ± 1.60 *	0.70		0.015	
35 day	28.00 ± 6.29	25.33 ± 5.99	1.74		0.469	
PCT (L/L)				<0.001	0.357	0.741
21 day	0.012 ± 0.008	0.011 ± 0.005	0.002		0.666	
35 day	0.032 ± 0.007	0.029 ± 0.007	0.002		0.402	
MPV (fL)				0.003	0.062	0.999
21 day	11.25 ± 0.10	11.08 ± 0.23	0.06		0.139	
35 day	11.53 ± 0.23	11.37 ± 0.23	0.07		0.237	
PDW (%)				0.081	0.491	0.589
21 day	70.02 ± 14.66	76.83 ± 14.91	4.20		0.443	
35 day	63.00 ± 11.46	63.83 ± 11.98	3.22		0.904	

* Represents a significant difference from CON (*p* < 0.05). ** Represents a significant difference from CON (*p* < 0.01). MCV: Mean corpuscular volume; MCH: mean corpuscular hemoglobin; MCHC: mean corpuscular hemoglobin concentration; RDW-SD: red blood cell distribution width—standard deviation; RDW-CV: red blood cell distribution width—variation coefficient; PCT: personal carbon trading; MPV: mean platelet volume; PDW: platelet distribution width. CON: control. DHF: designed herbal formula.

## Data Availability

Data are contained within the article.

## Supplementary Materials

The following supporting information can be downloaded at: https://www.mdpi.com/article/10.3390/vetsci11030107/s1, Table S1: Ingredients and nutrient composition of the basal diet.

## References

[1] Ayalew H., Zhang H., Wang J., Wu S., Qiu K., Qi G., Tekeste A., Wassie T., Chanie D. (2022). Potential feed additives as antibiotic alternatives in broiler production. Front. Vet. Sci..

[2] Zhu Q., Sun P., Zhang B., Kong L., Xiao C., Song Z. (2021). Progress on gut health maintenance and antibiotic alternatives in broiler chicken production. Front. Nutr..

[3] Chattopadhyay M.K. (2014). Use of antibiotics as feed additives: A burning question. Front. Microbiol..

[4] Liu Y., Wu Y., Wu J., Li X., Yu L., Xie K., Zhang M., Ren L., Ji Y., Li Y. (2022). Exposure to veterinary antibiotics via food chain disrupts gut microbiota and drives increased *Escherichia coli* virulence and drug resistance in young adults. Pathogens.

[5] Puvača N., Ljubojević Pelić D., Pelić M., Bursić V., Tufarelli V., Piemontese L., Vuković G. (2023). Microbial resistance to antibiotics and biofilm formation of bacterial isolates from different carp species and risk assessment for public health. Antibiotics.

[6] Tan M.F., Li H.Q., Yang Q., Zhang F.F., Tan J., Zeng Y.B., Wei Q.P., Huang J.N., Wu C.C., Li N. (2023). Prevalence and antimicrobial resistance profile of bacterial pathogens isolated from poultry in Jiangxi Province, China from 2020 to 2022. Poult. Sci..

[7] Mohammadagheri N., Najafi R., Najafi G. (2016). Effects of dietary supplementation of organic acids and phytase on performance and intestinal histomorphology of broilers. Vet. Res. Forum..

[8] Giannenas I., Papadopoulos E., Tsalie E., Triantafillou E., Henikl S., Teichmann K., Tontis D. (2012). Assessment of dietary supplementation with probiotics on performance, intestinal morphology and microflora of chickens infected with *Eimeria tenella*. Vet. Parasitol..

[9] Reda F.M., El-Saadony M.T., El-Rayes T.K., Farahat M., Attia G., Alagawany M. (2021). Dietary effect of licorice (*Glycyrrhiza glabra*) on quail performance, carcass, blood metabolites and intestinal microbiota. Poult. Sci..

[10] Kiarie E.G., Leung H., Akbari Moghaddam Kakhki R., Patterson R., Barta J.R. (2019). Utility of feed enzymes and yeast derivatives in ameliorating deleterious effects of coccidiosis on intestinal health and function in broiler chickens. Front. Vet. Sci..

[11] Mahmoud M.M., Al-Hejin A.M., Abujamel T.S., Ghetas A.M., Yacoub H.A. (2023). Chicken *β*-defensin-1 peptide as a candidate anticoccidial agent in broiler chickens. Anim. Biotechnol..

[12] Ogbuewu I.P., Okoro V.M., Mbajiorgu C.A. (2020). Meta-analysis of the influence of phytobiotic (pepper) supplementation in broiler chicken performance. Trop. Anim. Health Prod..

[13] Chen Z., Liu L., Gao C., Chen W., Vong C.T., Yao P., Yang Y., Li X., Tang X., Wang S. (2020). *Astragali radix* (Huangqi): A promising edible immunomodulatory herbal medicine. J. Ethnopharmacol..

[14] Zhao X., Sun W., Zhang S., Meng G., Qi C., Fan W., Wang Y., Liu J. (2016). The immune adjuvant response of polysaccharides from *Atractylodis macrocephalae* Koidz in chickens vaccinated against Newcastle disease (ND). Carbohydr. Polym..

[15] Wang H., Zheng X., Lin Y., Zheng X., Yan M., Li Y., Shi D., Guo S., Liu C. (2023). The mixture of *Radix isatidis*, *Forsythiae*, and *Gypsum alleviates* lipopolysaccharide-induced fever in broilers by inhibition of TLR4/NF-κB signaling pathway. Poult. Sci..

[16] Jiang X.-R., Zhang H.-J., Wang J., Wu S.-G., Yue H.-Y., Lü H.-Y., Cui H., Bontempo V., Qi G.-H. (2016). Effect of dried *Tangerine peel* extract supplementation on the growth performance and antioxidant status of broiler chicks. Ital. J. Anim. Sci..

[17] Xiao Y.Q., Shao D., Sheng Z.W., Wang Q., Shi S.R. (2019). A mixture of daidzein and Chinese herbs increases egg production and eggshell strength as well as blood plasma Ca, P, antioxidative enzymes, and luteinizing hormone levels in post-peak, brown laying hens. Poult. Sci..

[18] Li X.L., He W.L., Wang Z.B., Xu T.S. (2016). Effects of Chinese herbal mixture on performance, egg quality and blood biochemical parameters of laying hens. J. Anim. Physiol. Anim. Nutr..

[19] Fu G., Zhou Y., Song Y., Liu C., Hu M., Xie Q., Wang J., Zhang Y., Shi Y., Chen S. (2023). The effect of combined dietary supplementation of herbal additives on carcass traits, meat quality, immunity and cecal microbiota composition in Hungarian white geese. PeerJ.

[20] Wang C., Zhang T., Cui X., Li S., Zhao X., Zhong X. (2013). Hepatoprotective effects of a chinese herbal formula, longyin decoction, on carbon-tetrachloride-induced liver injury in chickens. Evid. Based Complement. Alternat. Med..

[21] Cheng Y., Geng Z., Li Y., Song X., Li L., Wen A., Yin Z. (2023). Effects of “Shi Ying Zi” powder and osthole on immune and antioxidant function of *Eimeria tenella*-infected broilers. Exp. Parasitol..

[22] Dale N. (1994). National Research Council Nutrient Requirements of Poultry-Ninth Revised Edition (1994). J. Appl. Poult. Res..

[23] Yang L., Chen L., Zheng K., Ma Y.J., He R.X., Arowolo M.A., Zhou Y.J., Xiao D.F., He J.H. (2022). Effects of fenugreek seed extracts on growth performance and intestinal health of broilers. Poult. Sci..

[24] (2019). Operating Procedure of Livestock and Poultry Slaughtering—Chicken.

[25] Martin M. (2011). Cutadapt removes adapter sequences from high-throughput sequencing reads. EMBnet. J..

[26] Edgar R.C., Haas B.J., Clemente J.C., Quince C., Knight R. (2011). UCHIME improves sensitivity and speed of chimera detection. Bioinformatics.

[27] Edgar R.C. (2013). UPARSE: Highly accurate OTU sequences from microbial amplicon reads. Nat. Methods.

[28] Quast C., Pruesse E., Yilmaz P., Gerken J., Schweer T., Yarza P., Peplies J., Glöckner F.O. (2013). The SILVA ribosomal RNA gene database project: Improved data processing and web-based tools. Nucleic Acids Res..

[29] Bolyen E., Rideout J.R., Dillon M.R., Bokulich N.A., Abnet C.C., Al-Ghalith G.A., Alexander H., Alm E.J., Arumugam M., Asnicar F. (2019). Reproducible, interactive, scalable and extensible microbiome data science using QIIME 2. Nat. Biotechnol..

[30] Rohart F., Gautier B., Singh A., Ka L.C. (2017). mixOmics: An R package for ‘omics feature selection and multiple data integration. PLoS Comput. Biol..

[31] Segata N., Izard J., Waldron L., Gevers D., Miropolsky L., Garrett W.S., Huttenhower C. (2011). Metagenomic biomarker discovery and explanation. Genome Biol..

[32] Cheng G., Hao H., Xie S., Wang X., Dai M., Huang L., Yuan Z. (2014). Antibiotic alternatives: The substitution of antibiotics in animal husbandry?. Front. Microbiol..

[33] Lee M., Shin H., Park M., Kim A., Cha S., Lee H. (2022). Systems pharmacology approaches in herbal medicine research: A brief review. BMB Rep..

[34] Liang X., Yamazaki K., Kamruzzaman M., Bi X., Panthee A., Sano H. (2013). Effects of Chinese herbal medicine on plasma glucose, protein and energy metabolism in sheep. J. Anim. Sci. Biotechnol..

[35] Lipiński K., Antoszkiewicz Z., Kotlarczyk S., Mazur-Kuśnirek M., Kaliniewicz J., Makowski Z. (2019). The effect of herbal feed additive on the growth performance, carcass characteristics and meat quality of broiler chickens fed low-energy diets. Arch. Anim. Breed..

[36] Oloruntola O.D., Agbede J.O., Ayodele S.O., Oloruntola D.A. (2019). Neem, pawpaw and bamboo leaf meal dietary supplementation in broiler chickens: Effect on performance and health status. J. Food Biochem..

[37] Behboodi H.R., Hosseini D., Salarieh A., Gholampour M., Panahi M., Alemi M., Baradaran A., Nazarpak H.H. (2022). Impact of drinking water supplementation of a blend of peppermint, coneflower (*Echinacea purpurea*), thyme, propolis, and prebiotic on performance, serum constituents, and immunocompetence of broiler chickens. Trop. Anim. Health Prod..

[38] Abd El-Hack M.E., El-Saadony M.T., Elbestawy A.R., Gado A.R., Nader M.M., Saad A.M., El-Tahan A.M., Taha A.E., Salem H.M., El-Tarabily K.A. (2022). Hot red pepper powder as a safe alternative to antibiotics in organic poultry feed: An updated review. Poult. Sci..

[39] Tufarelli V., Ghavami N., Nosrati M., Rasouli B., Kadim I.T., Suárez Ramírez L., Gorlov I., Slozhenkina M., Mosolov A., Seidavi A. (2023). The effects of peppermint (*Mentha piperita* L.) and chicory (*Cichorium intybus* L.) in comparison with a prebiotic on productive performance, blood constituents, immunity and intestinal microflora in broiler chickens. Anim. Biotechnol..

[40] Lin P.-H., Chen Z.-W., Liu J.-Y., Ye J.-C. (2023). Dietary supplementation of *Ocimum gratissimum* improves growth performance and immune response in broilers under high ambient temperature. J. Anim. Sci..

[41] Alagawany M., Elnesr S.S., Farag M.R., Abd El-Hack M.E., Khafaga A.F., Taha A.E., Tiwari R., Yatoo M.I., Bhatt P., Marappan G. (2019). Use of Licorice (*Glycyrrhiza glabra*) herb as a feed additive in poultry: Current knowledge and prospects. Animals.

[42] Yuan S., Wang Q., Li J., Xue J.C., Li Y., Meng H., Hou X.T., Nan J.X., Zhang Q.G. (2022). Inflammatory bowel disease: An overview of Chinese herbal medicine formula-based treatment. Chin. Med..

[43] Liu Y., Liang S., Zi X., Yan S., Liu M., Li M., Zhao Y., Dou T., Ge C., Wang K. (2022). Influence of Chinese herbal formula on bone characteristics of cobb broiler chickens. Genes.

[44] Ghafouri S.A., Ghaniei A., Tamannaei A.E.T., Sadr S., Charbgoo A., Ghiassi S., Abuali M. (2023). Evaluation of therapeutic effects of an herbal mixture (*Echinacea purpurea* and *Glycyrrhiza glabra*) for treatment of clinical coccidiosis in broilers. Vet. Med. Sci..

[45] Travel A., Petit A., Barat P., Collin A., Bourrier-Clairat C., Pertusa M., Skiba F., Crochet S., Cailleau-Audouin E., Chartrin P. (2021). Methodologies to assess the bioactivity of an herbal extract on immunity, health, welfare and production performance in the chicken: The case of *Melissa officinalis* L. extract. Front. Vet. Sci..

[46] Zhang L., Zhang R., Jia H., Zhu Z., Li H., Ma Y. (2021). Supplementation of probiotics in water beneficial growth performance, carcass traits, immune function, and antioxidant capacity in broiler chickens. Open Life Sci..

[47] Sławińska A., Siwek M., Zylińska J., Bardowski J., Brzezińska J., Gulewicz K.A., Nowak M., Urbanowski M., Płowiec A., Bednarczyk M. (2014). Influence of synbiotics delivered in ovo on immune organs development and structure. Folia Biol..

[48] Sjofjan O., Adli D.N., Harahap R.P., Jayanegara A., Utama D.T., Seruni A.P. (2021). The effects of lactic acid bacteria and yeasts as probiotics on the growth performance, relative organ weight, blood parameters, and immune responses of broiler: A meta-analysis. F1000Research.

[49] Li S., Ren L., Zhu X., Li J., Zhang L., Wang X., Gao F., Zhou G. (2019). Immunomodulatory effect of γ-irradiated *Astragalus* polysaccharides on immunosuppressed broilers. Anim. Sci. J..

[50] Zhou Y., Mao S., Zhou M. (2019). Effect of the flavonoid baicalein as a feed additive on the growth performance, immunity, and antioxidant capacity of broiler chickens. Poult. Sci..

[51] Song Z., Xie K., Zhang Y., Xie Q., He X., Zhang H. (2021). Effects of dietary ginsenoside Rg1 supplementation on growth performance, gut health, and serum immunity in broiler chickens. Front. Nutr..

[52] Abdel-Maksoud E.M., Daha A., Taha N.M., Lebda M.A., Sadek K.M., Alshahrani M.Y., Ahmed A.E., Shukry M., Fadl S.E., Elfeky M. (2023). Effects of ginger extract and/or propolis extract on immune system parameters of vaccinated broilers. Poult. Sci..

[53] Diaz Carrasco J.M., Casanova N.A., Fernández Miyakawa M.E. (2019). Microbiota, gut health and chicken productivity: What is the connection?. Microorganisms.

[54] Wickramasuriya S.S., Park I., Lee K., Lee Y., Kim W.H., Nam H., Lillehoj H.S. (2022). Role of physiology, immunity, microbiota, and infectious diseases in the gut health of poultry. Vaccines.

[55] Alharthi A.S., Alruwaili N.W., Al-Baadani H.H., Al-Garadi M.A., Shamlan G., Alhidary I.A. (2023). Investigating the effect of *Pulicaria jaubertii* as a natural feed additive on the growth performance, blood biochemistry, immunological response, and cecal microbiota of broiler chickens. Animals.

[56] Khan S., Moore R.J., Stanley D., Chousalkar K.K. (2020). The gut microbiota of laying hens and its manipulation with prebiotics and probiotics to enhance gut health and food safety. Appl. Environ. Microbiol..

[57] Zafar H., Saier M.H. (2021). Gut Bacteroides species in health and disease. Gut Microbes.

[58] Jandhyala S.M., Talukdar R., Subramanyam C., Vuyyuru H., Sasikala M., Nageshwar Reddy D. (2015). Role of the normal gut microbiota. World J. Gastroenterol..

[59] Luo Y.H., Peng H.W., Wright A.D., Bai S.P., Ding X.M., Zeng Q.F., Li H., Zheng P., Su Z.W., Cui R.Y. (2013). Broilers fed dietary vitamins harbor higher diversity of cecal bacteria and higher ratio of *Clostridium*, *Faecalibacterium*, and *Lactobacillus* than broilers with no dietary vitamins revealed by 16S rRNA gene clone libraries. Poult. Sci..

[60] Braz V.S., Melchior K., Moreira C.G. (2020). *Escherichia coli* as a multifaceted pathogenic and versatile bacterium. Front. Cell Infect. Microbiol..

[61] Kim J.S., Lee M.S., Kim J.H. (2020). Recent updates on outbreaks of shiga toxin-producing *Escherichia coli* and its potential reservoirs. Front. Cell Infect. Microbiol..

[62] Mirsepasi-Lauridsen H.C., Vallance B.A., Krogfelt K.A., Petersen A.M. (2019). *Escherichia coli* pathobionts associated with inflammatory bowel disease. Clin. Microbiol. Rev..

[63] Song B., Li P., Yan S., Liu Y., Gao M., Lv H., Lv Z., Guo Y. (2022). Effects of dietary Astragalus polysaccharide supplementation on the Th17/Treg balance and the gut microbiota of broiler chickens challenged with necrotic enteritis. Front. Immunol..

[64] Wang C., Liu Q., Ye F., Tang H., Xiong Y., Wu Y., Wang L., Feng X., Zhang S., Wan Y. (2021). Dietary Purslane (*Portulaca oleracea* L.) promotes the growth performance of broilers by modulation of gut microbiota. AMB Express.

[65] Guo L., He J., Zhang J., Zhang X., Zhang D., Zhou L., Yuan Y., Fu S., Qiu Y., Ye C. (2021). Baicalin-aluminum modulates the broiler gut microbiome. DNA Cell Biol..

[66] Wu S., Li T., Niu H., Zhu Y., Liu Y., Duan Y., Sun Q., Yang X. (2019). Effects of glucose oxidase on growth performance, gut function, and cecal microbiota of broiler chickens. Poult. Sci..

[67] Wang B., Gong L., Zhou Y., Tang L., Zeng Z., Wang Q., Zou P., Yu D., Li W. (2021). Probiotic *Paenibacillus polymyxa* 10 and *Lactobacillus plantarum* 16 enhance growth performance of broilers by improving the intestinal health. Anim. Nutr..

[68] Grondin J.M., Déjean G., Van Petegem F., Brumer H. (2022). Cell surface xyloglucan recognition and hydrolysis by the human gut commensal *Bacteroides uniformis*. Appl. Environ. Microbiol..

[69] Medina-Larqué A.S., Rodríguez-Daza M.C., Roquim M., Dudonné S., Pilon G., Levy É., Marette A., Roy D., Jacques H., Desjardins Y. (2022). Cranberry polyphenols and agave agavins impact gut immune response and microbiota composition while improving gut barrier function, inflammation, and glucose metabolism in mice fed an obesogenic diet. Front. Immunol..

[70] Vallianou N.G., Kounatidis D., Tsilingiris D., Panagopoulos F., Christodoulatos G.S., Evangelopoulos A., Karampela I., Dalamaga M. (2023). The role of next-generation probiotics in obesity and obesity-associated disorders: Current knowledge and future perspectives. Int. J. Mol. Sci..

[71] Ferreira-Halder C.V., Faria A.V.S., Andrade S.S. (2017). Action and function of *Faecalibacterium prausnitzii* in health and disease. Best Pract. Res. Clin. Gastroenterol..

[72] Sokol H., Pigneur B., Watterlot L., Lakhdari O., Bermúdez-Humarán L.G., Gratadoux J.J., Blugeon S., Bridonneau C., Furet J.P., Corthier G. (2008). *Faecalibacterium prausnitzii* is an anti-inflammatory commensal bacterium identified by gut microbiota analysis of Crohn disease patients. Proc. Natl. Acad. Sci. USA.

[73] Miquel S., Leclerc M., Martin R., Chain F., Lenoir M., Raguideau S., Hudault S., Bridonneau C., Northen T., Bowen B. (2015). Identification of metabolic signatures linked to anti-inflammatory effects of *Faecalibacterium prausnitzii*. mBio.

[74] De Cesare A., Sala C., Castellani G., Astolfi A., Indio V., Giardini A., Manfreda G. (2020). Effect of *Lactobacillus acidophilus* D2/CSL (CECT 4529) supplementation in drinking water on chicken crop and caeca microbiome. PLoS ONE.

[75] Shad A.A., Shad W.A. (2021). *Shigella sonnei*: Virulence and antibiotic resistance. Arch. Microbiol..

[76] Anokwuru C.P., Tankeu S., van Vuuren S., Viljoen A., Ramaite I.D.I., Taglialatela-Scafati O., Combrinck S. (2020). Unravelling the antibacterial activity of *Terminalia sericea* Root bark through a metabolomic approach. Molecules.

