# Supplementary material for: Effects of Designed Herbal Formula on Growth Performance, Blood Indices, Organ Traits, and Cecum Microbiology in Broilers
Source: Vet Sci. 2024 Feb 29;11(3):107. doi: 10.3390/vetsci11030107 (PMC10975483; doi:10.3390/vetsci11030107)
Supplement: Supplementary file 1 [file vetsci-11-00107-s001.zip › vetsci-2829559-supplementary.pdf]

Table S1. Ingredients and nutrient composition of the basal diet.

Ingredient	CON	0.5%DHF	1.0%DHF	1.5%DHF
Corn (%)	54.75	54.25	53.75	53.25
Soybean meal (%)	37.00	37.00	37.00	37.00
Soya bean oil (%)	4.10	4.10	4.10	4.10
Calcium hydrogen phosphate (%)	1.35	1.35	1.35	1.35
Stone powder (%)	1.15	1.15	1.15	1.15
DHF (%)	-	0.50	1.00	1.50
Salt (%)	0.30	0.30	0.30	0.30
Methionine (%)	0.20	0.20	0.20	0.20
Lysine (%)	0.15	0.15	0.15	0.15
Premix[1] (%)	1.00	1.00	1.00	1.00
Total (%)	100.00	100.00	100.00	100.00
Metabolisable energy (kcal/kg)	3002.00	2986.68	2971.36	2956.04
Crude protein (%)	21.50	21.49	21.49	21.48
Lysine (%)	1.27	1.27	1.27	1.27
Methionine (%)	0.51	0.51	0.51	0.51
Tryptophan (%)	0.25	0.25	0.25	0.25
Arginine (%)	1.46	1.46	1.46	1.46
Threonine (%)	0.80	0.80	0.80	0.80
Valine (%)	0.98	0.98	0.98	0.98
Isoleucine (%)	0.87	0.87	0.87	0.87
Leucine (%)	1.75	1.75	1.74	1.74
Calcium (%)	1.00	1.00	1.00	1.00
Total phosphorus (%)	0.68	0.68	0.68	0.68
Non-phytate phosphorus (%)	0.45	0.45	0.45	0.45

[1] Premix provided the following per kilogram of diets：VA 10 000.00 IU，VB_1 2.00 mg，VB_2 8.00 mg，VB_6 4.00 mg，VB_{12} 0.06 mg，VD_3 3 000.00 IU，VE 20.00 IU，VK_3 2.00 mg，biotin 0.20 mg，folic acid 1.00 mg， *D*-pantothenic acid 20.00 mg，nicotinic acid 50.0 mg，Cu (as copper sulfate) 10.00 mg，Fe (as ferrous sulfate) 60.00 mg，Mn (as manganese sulfate) 80.00 mg，Zn (as zinc sulfate) 60.00 mg，I (as potassium iodide) 0.20 mg，Se (as sodium selenite) 0.30 mg，choline 1000 mg/kg.
